# Radical Treatment for Prostate Cancer in Men With Limited Life Expectancy in Sweden

**DOI:** 10.1001/jamanetworkopen.2025.8572

**Published:** 2025-05-06

**Authors:** Eugenio Ventimiglia, Rolf Gedeborg, Johan Styrke, Andri Wilberg Orrason, Pär Stattin, Marcus Westerberg

**Affiliations:** 1 Department of Surgical Sciences, Uppsala University, Uppsala, Sweden; 2 Department of Diagnostics and Intervention, Urology and Andrology, Umeå University, Umeå, Sweden

## Abstract

This cohort study compares rates of radical treatment for prostate cancer, despite current guideline recommendations that discourage its use for men with limited life expectancy, in Sweden and the US.

## Introduction

Current guidelines in Sweden and the US discourage the use of radical treatment for men with prostate cancer who have limited life expectancy.^1^ However, Daskivich et al^2^ recently reported that despite these recommendations, a large proportion of men with prostate cancer and limited life expectancy in a US Department of Veterans Affairs (VA) health system cohort received radical treatment. Motivated by these findings, we investigated the use of radical treatment for prostate cancer according to risk category and life expectancy in Sweden, where a tax-funded national health care system delivers care to all citizens at reasonable cost.

## Methods

This cohort study used data from the National Prostate Cancer Register (NPCR), which captures 98% of all incident cases of prostate cancer in Sweden. In the Prostate Cancer Database Sweden, NPCR is linked to other national health care registers and demographic databases. The Swedish Research Ethics Authority reviewed and approved the study and waived the need for informed consent as NPCR has an opt-out option. The study followed the STROBE reporting guideline.

We extracted data on all men, irrespective of age, with localized or locally advanced prostate cancer between 2007 and 2023. Life expectancy at the date of diagnosis was estimated as described previously,^3^ and we used a modification of the National Comprehensive Cancer Network risk categorization outlined in the Table. Within each subgroup of life expectancy and risk category, we calculated the crude proportion of men who received radical prostatectomy or radical radiotherapy within 1 year from diagnosis and the annual trend based on a logistic regression model. Data on the proportion of VA men receiving radical treatment for prostate cancer were manually extracted from Daskivich et al^2^ and plotted in the Figure. All statistical analyses were performed using R, version 4.0.4 (R Foundation). *P* < .05 was set as the threshold for significance.

**Table.** Baseline Characteristics of Men With Localized or Locally Advanced Prostate Cancer in the National Prostate Cancer Register of Sweden According to Life Expectancy

Characteristic	Patients, No. (%)		
	Life expectancy ≥10 y (n = 121 173)	Life expectancy <10 y (n = 29 157)	Life expectancy <5 y (n = 5896)
Age at diagnosis, y			
Median (IQR)	67 (62-72)	81 (77-84)	85 (80-88)
<65	16 682 (13.8)	44 (0.2)	7 (0.1)
60-69	53 957 (44.5)	1145 (3.9)	113 (1.9)
70-79	47 176 (38.9)	8812 (30.2)	934 (15.8)
≥80	3358 (2.8)	19 156 (65.7)	4842 (82.1)
Life expectancy, y^a^			
Median (IQR)	18 (14-22)	7 (5-9)	4 (3-5)
Year of diagnosis^b^			
2007-2012	43 397 (32.6)	12 280 (33.3)	2772 (33.5)
2013-2017	40 676 (30.6)	10 398 (28.2)	2456 (29.7)
2018-2023	48 829 (36.7)	14 246 (38.6)	3051 (36.9)
Risk category^c^			
Low risk	31 653 (26.1)	1899 (6.5)	206 (3.5)
Favorable intermediate risk	31 788 (26.2)	3586 (12.3)	452 (7.7)
Unfavorable intermediate risk	28 569 (23.6)	5899 (20.2)	815 (13.8)
High risk	29 163 (24.1)	17 773 (61.0)	4423 (75.0)
Treatment			
Radical prostatectomy	42 290 (34.9)	622 (2.1)	24 (0.4)
Radical radiotherapy	26 304 (21.7)	3169 (10.9)	139 (2.4)
No radical treatment	52 579 (43.4)	25 366 (87.0)	5733 (97.2)

^a^ Life expectancy was computed using a previously described method^3^ in which comorbidity was measured by the Multidimensional Diagnosis-Based Comorbidity Index based on diagnoses in the Patient Registry and the Drug Comorbidity Index based on dispensations in the Prescribed Drug Registry.^3,4^

^b^ Percentages were based on the number of patients without missing data on year of diagnosis.

^c^ Risk categories are according to the National Prostate Cancer Register, a modification of National Comprehensive Cancer Network risk categorization, as follows: low risk (cT1-2, Gleason score of 6, and prostate-specific antigen [PSA] <10 ng/mL), favorable intermediate risk (cT1-2, Gleason score of 7 [3 + 4], and PSA <10 ng/mL or Gleason score of 6 and PSA of 10-20 ng/mL), unfavorable intermediate risk (cT1-2, Gleason score of 7 [4 + 3], and PSA <10 ng/mL or Gleason score of 7 [3 + 4] and PSA of 10-20 ng/mL), and high risk (cT3-4, Gleason score of 8-10, and PSA ≥20 ng/mL).

**Figure.**
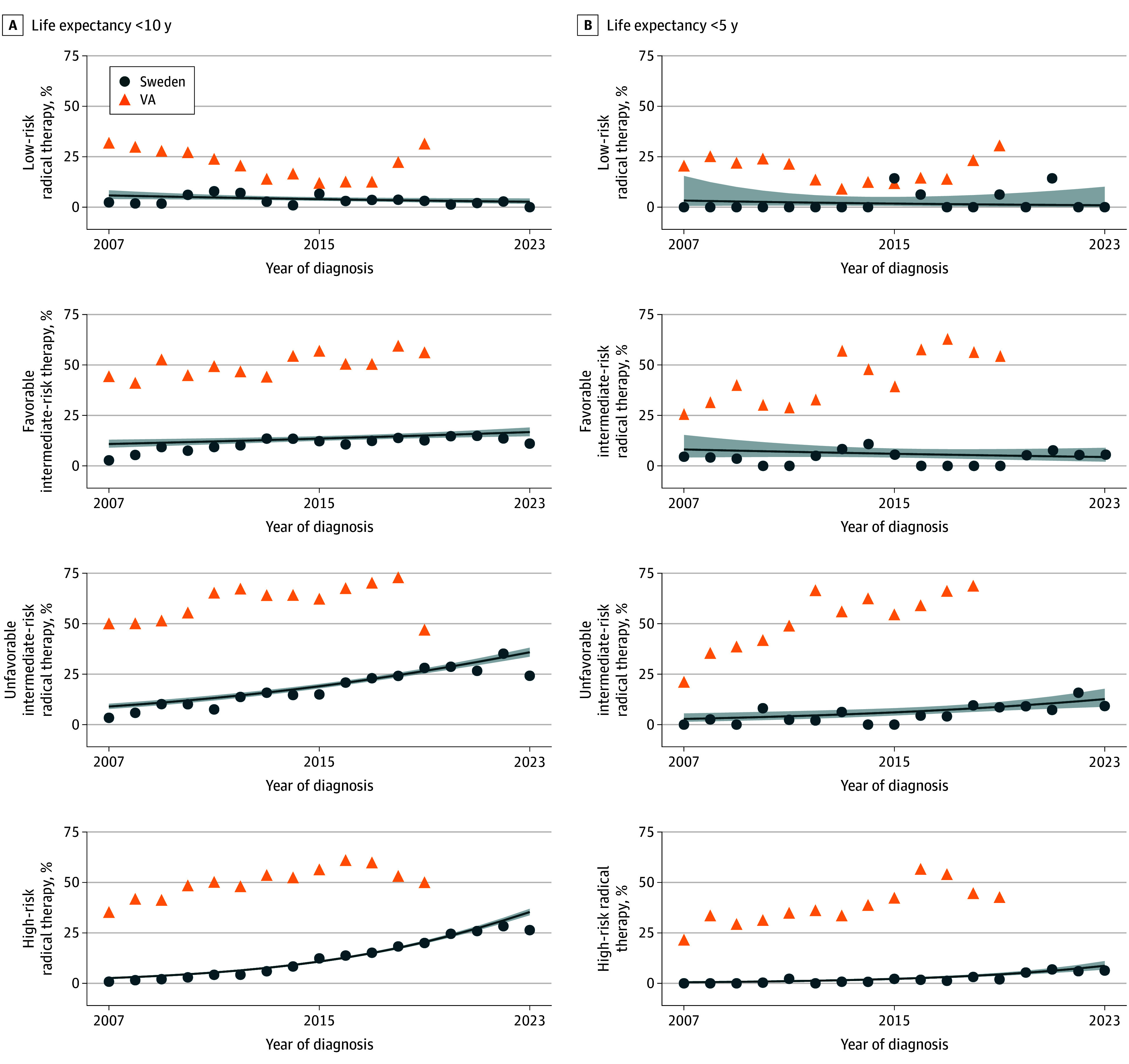
Proportion of Men With Localized and Locally Advanced Prostate Cancer in the National Prostate Cancer Register of Sweden and in the US Department of Veterans Affairs (VA) Health System Who Received Radical Therapy Swedish national guidelines for prostate cancer^1^ recommend against radical therapy in men with low-risk prostate cancer and men with favorable intermediate-risk prostate cancer with a life expectancy of 10 to 15 years or less and in men with high-risk prostate cancer with a life expectancy of less than 5 years. The linear annual trend was estimated using a logistic regression model within each subgroup. Life expectancy was computed using a previously described method^3^ in which comorbidity was measured by the Multidimensional Diagnosis-Based Comorbidity Index based on diagnoses in the Patient Registry and the Drug Comorbidity Index based on dispensations in the Prescribed Drug Registry.^3,4^

## Results

A total of 150 330 men with prostate cancer (median [IQR] age, 69 [63-75] years) fulfilled our inclusion criteria (Table). The proportion of men who received radical treatment by risk category and life expectancy strata is shown in the Figure. For men with life expectancy less than 10 years (median [IQR] age, 81 [77-84] years) for whom guidelines in both Sweden and the US recommend against radical therapy, a much higher proportion of VA men received radical therapy compared with men in Sweden. There was a slight increase in both health care systems during the study period, with radical treatment being as high as 26.4% in 2013 in the high-risk group in Sweden. In Sweden, the proportion of men with life expectancy less than 5 years (median [IQR] age, 85 [80-88] years) who underwent radical treatment was less than 16% in all risk categories.

## Discussion

This cohort study suggests that a low proportion of men with limited life expectancy diagnosed with localized or locally advanced prostate cancer received radical therapy in Sweden against guideline recommendations. These proportions were much lower than recently reported from the VA health system and other US health care databases.^4^ The difference in treatment strategy between these 2 equal-access health care systems is intriguing and warrants further investigation. We argue that public reporting of hospital-level data has contributed to adherence to guideline recommendations in Sweden,^1^ as previously shown for low-risk prostate cancer.^5,6^ However, inherent differences between these 2 populations, as well as differences in the methodology for estimating life expectancy in our study compared with the VA study, represent a limitation. We hope that our data will stimulate discussions about avoiding unnecessary, costly radical treatment with a high risk of adverse effects in men with limited life expectancy and diagnosed with prostate cancer.

## Notes

Supplement.
Data Sharing Statement
