# Error in Author Name

**DOI:** 10.1001/jamanetworkopen.2025.41880

**Published:** 2025-10-10

In the Research Letter titled “Radical Treatment for Prostate Cancer in Men With Limited Life Expectancy in Sweden,”^1^ published May 6, 2025, Dr Andri Wilberg Orrason’s name appeared incorrectly in the Article Information. This article has been corrected.^1^
