# Supplementary material for: Radical Treatment for Prostate Cancer in Men With Limited Life Expectancy in Sweden
Source: JAMA Netw Open. 2025 May 6;8(5):e258572. doi: 10.1001/jamanetworkopen.2025.8572 (PMC12056566; doi:10.1001/jamanetworkopen.2025.8572)
Supplement: Supplement. — Data Sharing Statement [file jamanetwopen-e258572-s001.pdf]

Data Sharing Statement

Ventimiglia. Radical Treatment for Prostate Cancer in Men With Limited Life Expectancy in Sweden. *JAMA Netw Open*. Published May 06, 2025.
doi:10.1001/jamanetworkopen.2025.8572

Data
Data available: No
